# Idiopathic Subglottic Stenosis: Improving Diagnosis and Awareness

**DOI:** 10.7759/cureus.109544

**Published:** 2026-05-24

**Authors:** Hannah E Phillips, Lee Reussner, Jennifer M Cannady

**Affiliations:** 1 Otolaryngology, Des Moines University, Des Moines, USA; 2 Otolaryngology, Lawrence Otolaryngology Associates, Lawrence, USA; 3 Speech-Language Pathology, Lawrence Otolaryngology Associates, Lawrence, USA

**Keywords:** diagnosis, flow volume loops, idiopathic subglottic stenosis, shortness of breath, stenosis, subglottic stenosis

## Abstract

Background

Idiopathic subglottic stenosis is a rare condition characterized by a constriction of the airway passage without a known cause. While there may be many contributing factors, no definitive etiology has been established. Due to its rarity, this condition is frequently overlooked by healthcare providers, resulting in an inaccurate and lengthy diagnosis process. This results in detrimental effects for the patient given the symptoms of progressive dyspnea. Our objective was to retrospectively review patients diagnosed with idiopathic subglottic stenosis and analyze key characteristics about each patient. By examining these cases in conjunction with the current literature, we hope to raise awareness of idiopathic subglottic stenosis and support an earlier accurate diagnosis. In particular, female patients with audible turbulent air flow on exam and a lack of response to bronchodilators should be promptly considered for a diagnosis of subglottic stenosis.

Methods

This study retrospectively reviews 10 patients, 18 years or older, diagnosed with idiopathic subglottic stenosis at an otolaryngology clinic in Kansas. Patients were evaluated first with a physical examination that included stroboscopy and flow volume loops. In addition, key variables analyzed included patients' age, gender, other diagnosis/treatment tried, length of time until the stenosis was diagnosed, base peak expiratory flow, symptoms, observed signs, treatment, and improvement after treatment. The data from these variables were then compiled into a table, and mean values were computed.

Results

In this study, all patients were Caucasian females between the ages of 26 and 78 years old, with a mean age of 46.7 years old. Ninety percent of the patients had been diagnosed previously with either asthma or chronic obstructive pulmonary disease (COPD) and were treated with corticosteroids as a result. The average time it took for these patients to be diagnosed from the onset of symptoms was approximately 36.8 months. The average base peak expiratory flow, gathered from pulmonary function testing, was 47% of the predicted value. They presented with a mixture of symptoms including dyspnea with exertion or at rest, stridor, a chronic cough, and dysphonia. In the office, the observed sign of turbulent airflow at rest was detected in nine out of the 10 patients. All patients underwent a biopsy, followed by laser excision of stenosis, steroid injection, and balloon dilation. All reported subjective relief in symptoms and, where available, showed objective improvement in pulmonary function testing and endoscopic appearance after the treatment.

Conclusions

A thorough history, a careful physical examination, pulmonary function testing, and visualization with endoscopy can significantly expedite the diagnosis of idiopathic subglottic stenosis. In addition, listening for abnormal upper airway sounds is crucial in guiding clinicians toward this diagnosis quickly.

## Introduction

Idiopathic subglottic stenosis is a condition where the trachea has decreased in size without a known reason. Only 18-33% of the subglottic stenosis cases are considered idiopathic [1,2] with an incidence of approximately 1/400,000 [3]. In comparison, iatrogenic subglottic stenosis occurs in 1/200,00 people annually [4]. While there are many causes of subglottic stenosis, a significant portion remain idiopathic [5]. The lack of information surrounding this condition can lead to incorrect or late diagnoses with possible life-threatening airway consequences. The purpose of this study is to evaluate our last 10 idiopathic subglottic stenosis patients with the goal of finding any common presenting symptoms and clinical patterns. Additionally, we aim to explore how this disease can be differentiated from other respiratory conditions. Through this series and a review of current literature, we seek to provide current knowledge about idiopathic subglottic stenosis and support earlier recognition and management by physicians and other providers, ultimately helping patients in a more timely and effective manner.

## Materials and methods

Study design

This was a retrospective observational study.

Study objective

The primary objective of this study was to assess and record the characteristics of idiopathic subglottic stenosis from the history, physical examination, nasolaryngoscopy, and pulmonary function tests. Overall, these features were analyzed to help clinicians diagnose this condition more accurately and efficiently.

Study setting

This study was conducted at Lawrence Otolaryngology Associates in Lawrence, Kansas. The same otolaryngologist performed the initial flexible laryngoscopy and also performed the direct microlaryngoscopy with biopsy, laser excision, injection, and balloon dilation. The biopsy results were analyzed by a board-certified pathology team from a single community hospital in Kansas.

Study duration

This study looked at the most recent consecutive patients with idiopathic subglottic stenosis over the past few years.

Study population

Medical records of the 10 most recent patients who received surgery for idiopathic subglottic stenosis were reviewed. Evaluation included a physical exam with stroboscopy and flow volume loops. The surgical management involved direct microlaryngoscopy with biopsy, laser excision, injection, and balloon dilation.

Inclusion criteria

Eligible participants were adult patients (18 years old or older) with shortness of breath and an office examination consistent with subglottic stenosis. Patients with subglottic stenosis were taken to surgery where a tissue biopsy was performed and analyzed. The biopsy results of each of these patients revealed inflammation and fibrosis. There was no evidence of malignancy, vasculitis, or granulomas noted on biopsy. These patients were then diagnosed with idiopathic subglottic stenosis and included in our study. While we have seen patients who had been diagnosed and treated for subglottic stenosis in the past, we only included those who had no prior diagnosis or treatment of subglottic stenosis. In addition, initial flow volume loops and a video nasolaryngoscopy examination were required. Some patients obtained pulmonary function testing at an outside site where the peak expiratory flow was not computed.

Exclusion criteria

Patients were excluded from the study if they were younger than 18 years old or had a biopsy done at the time of surgery that revealed granulomas, malignancy, or other pathology that was not consistent with an idiopathic-based stenosis. No patients were included if they had an intubation within the previous six months. In addition, if a patient did not have an initial video nasolaryngoscopy or a flow volume loop performed, they were excluded from the study.

Study protocol and data analysis

A variety of data was collected on each patient including the patient's age, gender, symptoms, previous assigned misdiagnoses, observed signs, base peak expiratory flow (percent of predicted) from the flow volume loop, time from the onset of symptoms until the stenosis was diagnosed (months), and the improvement after treatment. The data was then compiled into a table form with mean values calculated for the appropriate data.

## Results

We reviewed the last 10 patients with idiopathic subglottic stenosis at our office. The patients' age, gender, other diagnosis/treatment tried, length of time until the stenosis was diagnosed, base peak expiratory flow, symptoms, observed signs, treatment, and improvement after treatment were analyzed. All patients underwent biopsy, laser excision, steroid injection, and balloon dilation, and those with recorded data reported significant subjective relief in symptoms and objective improvement in pulmonary function testing after the treatment. The data is presented in Table 1.

**Table 1:** Review of 10 patients diagnosed with idiopathic subglottic stenosis

	Age (years)	Gender	Symptoms	Other diagnoses originally given	Observed signs	Base peak expiratory flow (percent of predicted) from the flow volume loop	Time from the onset of symptoms until the stenosis was diagnosed (months)	Improvement after treatment (all 10 patients felt subjectively improved)
Patient 1	42	Female	Difficulty breathing, unable to catch breath, and voice seemed less smooth	Allergic rhinitis	Audible turbulent airflow on inspiration, even at rest. Flexible nasolaryngoscopy showed a 75% circumferential narrowing 3 cm below the vocal folds	37% of predicted. Moderate flattening of inspiratory and expiratory loops	18 months	A flexible fiberoptic laryngoscopy was performed six months post-op and showed an airway that was patent and an area that revealed 10% stenotic, though it was not affecting her airflow. Pulmonary function tests were not performed
Patient 2	78	Female	Upper airway noise, slight wheezing, shortness of breath	Asthma	Audible intermittent turbulent airflow on inspiration. Flexible nasolaryngoscopy and upper bronchoscopy showed a 60% circumferential narrowing 2 cm below the vocal folds	61% of predicted. Moderate flattening of the inspiratory loop and mild flattening of the expiratory loop	Weeks to months	During her postoperative visit, a flexible fiberoptic laryngoscopy revealed no stridor and an open airway. Pulmonary function tests were not performed
Patient 3	68	Female	Shortness of breath, which has limited her activity	Asthma	Audible turbulent airflow on inspiration, even at rest. Flexible nasolaryngoscopy showed a 75% narrowing about 2 cm below the vocal folds	45% of predicted. Moderate flattening of inspiratory and expiratory loops	12-24 months	The one-month postoperative visit showed the subglottic area to be dramatically improved and normal. Pulmonary function tests were not performed
Patient 4	38	Female	Progressive difficulty in breathing, shortness of breath on exertion, can hear herself breathing	Asthma	Audible turbulent airflow on inspiration with deep inspiration. Flexible nasolaryngoscopy showed a 75% narrowing 2 cm below the vocal folds	31% of predicted. Moderate flattening of inspiratory and expiratory loops	24 months	The three-month postoperative visit showed the subglottic area to be widely open. Peak expiratory flow 109% of predicted and a normal flow volume loop
Patient 5	26	Female	Wheezing, rhonchi, mild hoarseness	Asthma	Audible turbulent airflow on inspiration, even at rest. Flexible nasolaryngoscopy showed a 50% narrowing 2 cm below the vocal folds	43% of predicted. Moderate flattening of inspiratory and expiratory loops	48 months	Laryngeal stroboscopy showed dramatic improvement in the airway, and it looked to be 95% open. Pulmonary function tests were not performed
Patient 6	56	Female	Shortness of breath; difficult to walk due to breathing. Feels as if the throat is tight and the airway is constricted	Allergic rhinitis	Audible turbulent airflow noted. Flexible nasolaryngoscopy showed a 50% narrowing 2 cm below the vocal folds	No data available for peak expiratory flow. Mild flattening of inspiratory limbs	12-24 months	The two-week postoperative visit showed the subglottic area to be healing. Peak expiratory flow was 62% of predicted, and there was mild flattening of the flow volume loop
Patient 7	40	Female	Shortness of breath on exertion, shortness of breath when projecting her voice	Asthma	Communicates verbally with a normal voice; no data on turbulent airflow. Flexible nasolaryngoscopy showed a 50-60% circumferential narrowing 2 cm below the vocal folds	55% of predicted. Moderate flattening of inspiratory and expiratory loops	18 months	The two-week postoperative visit showed minimal subglottic stenosis. Peak expiratory flow 101% of predicted and a normal flow volume loop
Patient 8	31	Female	Ongoing cough, occasional wheezing with exertion, and a gurgling quality in her chest. Difficulty keeping up with others when hiking or walking briskly	Asthma	Audible turbulent airflow on inspiration. Flexible nasolaryngoscopy showed a 50-60% circumferential narrowing 2 cm below the vocal folds	64% of predicted. No visual records of a flow volume loop	120 months	The two- to three-week postoperative visit showed the subglottis to look better and open perhaps by another 50-60% from before surgery. Peak expiratory flow 83% of predicted and a normal flow volume loop
Patient 9	53	Female	Increased shortness of breath, difficulty breathing with exertion. Also feels voice is getting increasingly husky	Asthma and allergic rhinitis	Mild audible turbulent airflow on inspiration, even at rest. Flexible nasolaryngoscopy showed a 75% narrowing of the airway below the vocal folds	21% of predicted. Severe flattening of inspiratory and expiratory loops	96 months	Postoperatively, stroboscopy showed the subglottic area to be improved. Pulmonary function tests were not performed
Patient 10	35	Female	Shortness of breath	No previous diagnoses	Audible turbulent airflow on inspiration and expiration. A bronchoscopy was performed which showed a 60% or 70% narrowing 2 cm or so below the vocal folds	52% of predicted. Moderate flattening of inspiratory and expiratory loops	7 months	No current data on the patient's postoperative visits

**Figure 1:**
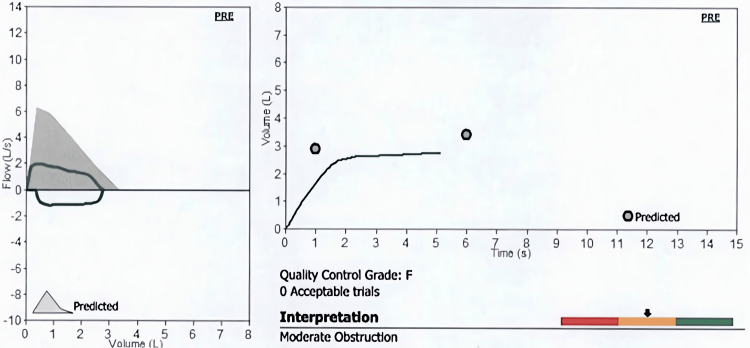
Preoperative flow volume loop data

Pulmonary function testing for patients with this condition typically demonstrated significantly flattened flow volume loops with a significantly decreased peak expiratory flow, illustrating a moderate obstruction (Figure 1).

In this particular patient example, the peak expiratory flow showed 31% of the predicted value (Table 2).

**Table 2:** Preoperative flow volume loop data including base peak expiratory flow FVC: forced vital capacity in liters (L); FEV1: forced expiratory volume in 1 second in liters (L); FEV1/FVC: ratio of forced expiratory volume in 1 second in liters to forced vital capacity in liters; PEF: peak expiratory flow in liters per second (L/s); ELA: estimated lung age in years; FEF2575: forced expiratory flow 25-75%; FET: forced expiratory time; F1VC: forced inspiratory vital capacity in liters (L); LLN: lower limit of normal; Pred: predicted value; Best: highest value achieved from all acceptable tests; %Pred: percent predicted; Z-score: number of standard deviations that a value is away from a predicted value; Pre #1: pre-bronchodilator, trial #1

Parameters	LLN	Pred	Best	%Pred	Z-score	Pre #1
FVC, L	2.61	3.4	2.78	82	-1.28	2.78
FEV1, L	2.23	2.87	1.77	62	-2.81	1.77
FEV1/FVC, %	72.6	84.3	63.7	76	-2.92	63.7
PEF, L/s	3.66	6.3	1.96	31	-2.71	1.96
ELA, years	-	38	96	253	-	96
FEF2575, L/s	1.91	3.31	1.59	48	-2.01	1.59
FET	-	6	5.1	85	-	5.1
F1VC, L	2.61	3.4	-	-	-	-

**Figure 2:**
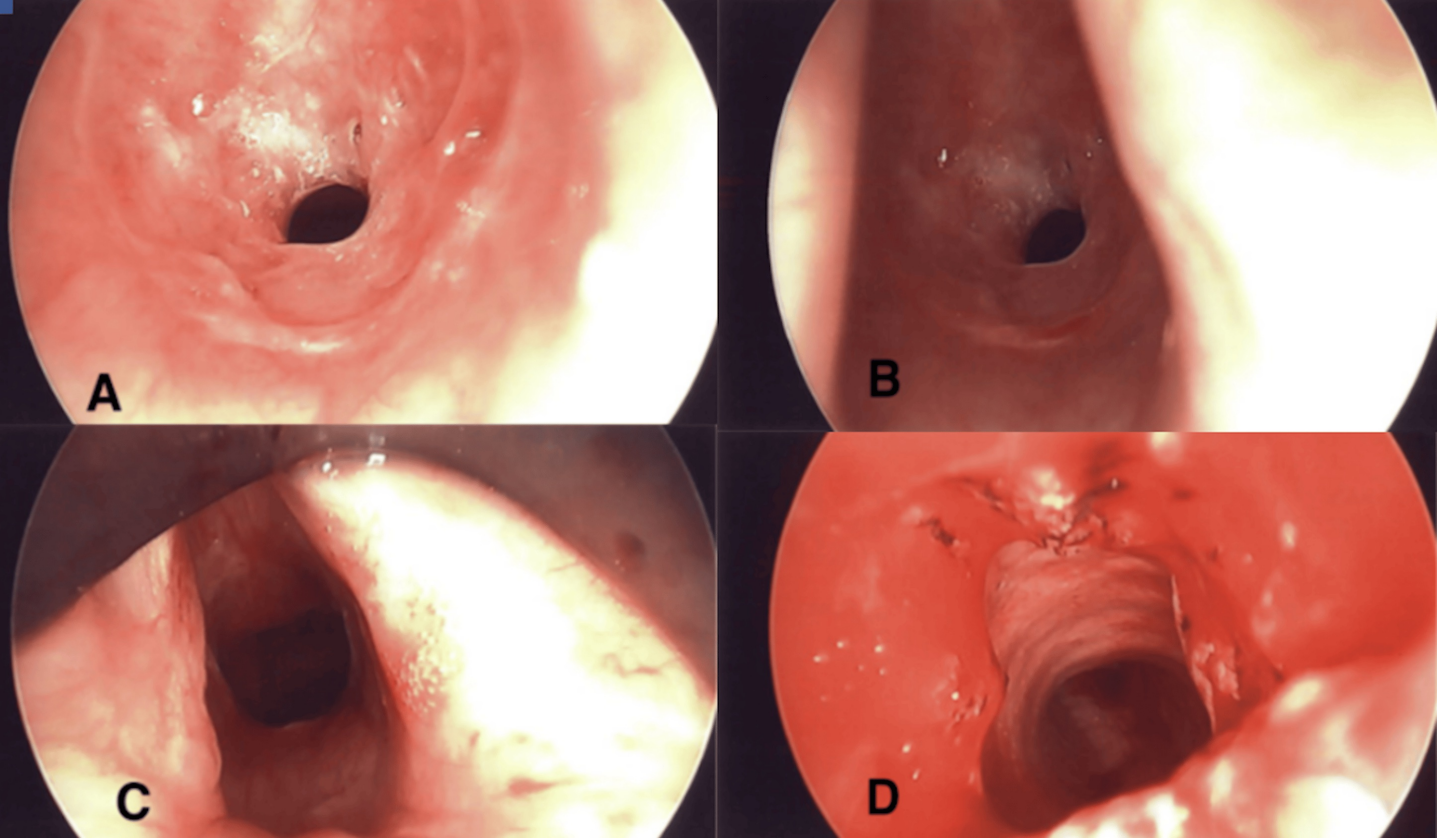
Intraoperative images. A and B show an area of stenosis, while C and D were taken following the laser excision and balloon dilation

A biopsy, steroid injection, laser excision, and balloon dilation were then performed for all of the 10 patients. Example intraoperative images taken before and after the relief of the stenosis are shown in Figure 2.

**Figure 3:**
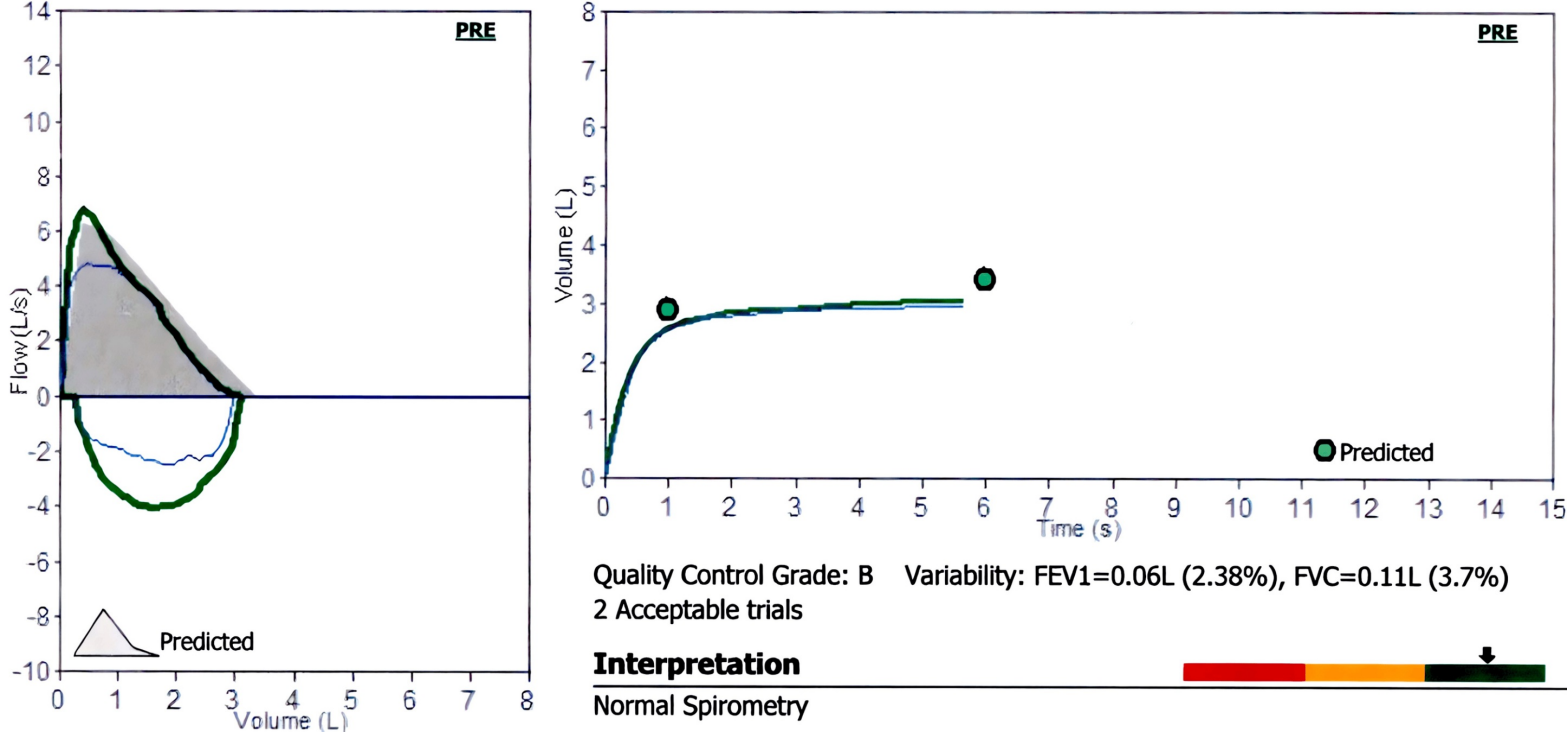
Postoperative flow volume loop data

Several months postoperatively, a repeat pulmonary function test can be performed. In this particular case, the flow volume loop was back to baseline and showed a normal spirometry (Figure 3).

The peak expiratory flow was at 109% of the predicted value, which was a significant improvement over the value of 31% of predicted before surgery (Table 3).

**Table 3:** Postoperative flow volume loop data including base peak expiratory flow FVC: forced vital capacity in liters (L); FEV1: forced expiratory volume in 1 second in liters (L); FEV1/FVC: ratio of forced expiratory volume in 1 second in liters to forced vital capacity in liters; PEF: peak expiratory flow in liters per second (L/s); ELA: estimated lung age in years; FEF2575: forced expiratory flow 25-75%; FET: forced expiratory time; F1VC: forced inspiratory vital capacity in liters (L); LLN: lower limit of normal; Pred: predicted value; Best: highest value achieved from all acceptable tests; %Pred: percent predicted; Z-score: number of standard deviations that a value is away from a predicted value; Pre #1: pre-bronchodilator, trial #1

Parameters	LLN	Pred	Best	%Pred	Z-score	Pre #1	Pre #2
FVC, L	2.61	3.4	3.08	91	-0.66	2.97	3.08
FEV1, L	2.23	2.87	2.58	90	-0.74	2.52	2.58
FEV1/FVC, %	72.6	84.3	83.8	99	-0.07	84.8	83.8
PEF, L/s	3.66	6.3	6.84	109	-0.34	4.85	6.84
ELA, years	-	38	53	139	-	56	53
FEF2575, L/s	1.91	3.31	2.89	87	-0.49	3	2.89
FET	-	6	5.61	94	-	5.64	5.61
F1VC, L	2.61	3.4	-	-	-	-	-
FEV1/FVC, %	72.6	84.3	-	-	-	-	-

## Discussion

Idiopathic subglottic stenosis is characterized by narrowing and inflammation of the upper airway [6]. Histologically, submucosal fibrosis is typically found in the lamina propria of the airway [7]. In most cases, the stenotic area begins at the cricoid cartilage and extends from the upper edge of the cricoid cartilage to the first tracheal ring. The narrowing is often circumferential, but could occasionally be eccentric. The affected segment typically measures between 1 and 2 cm [1], which aligns with our findings, in which most cases of stenosis had a length of approximately 1-1.5 cm. According to the current literature, this condition predominantly affects Caucasian women between the ages of 30 and 60 years old, with a mean age of 48.9 years old [8]. In our study, all patients were Caucasian females ranging in age from 26 to 78 years old, with a mean age of 46.7 years old.

Common causes of subglottic stenosis include iatrogenic factors, such as tracheotomies and intubations, autoimmune conditions including amyloidosis, sarcoidosis, and granulomatosis with polyangiitis, as well as trauma [1]. However, the cause of idiopathic stenosis remains elusive. Some authors have proposed that the development of idiopathic subglottic stenosis results from ongoing inflammation caused by an initial trigger [7]. Two potential triggers that are currently under investigation are estrogen and acid reflux. Given that idiopathic subglottic stenosis occurs almost exclusively in females, estrogen is suspected to play a role in its pathogenesis. However, current studies have not definitively established a causative factor in initiating the inflammatory response that leads to stenosis [7,9]. Ongoing research is exploring the role of various estrogen receptors and their potential impact on the trachea [7].

Extraesophageal reflux has also been proposed as a contributing factor in the development of idiopathic subglottic stenosis. Although research in this area is ongoing, a study by Blumin and Johnston found that more than half of the patients they analyzed with idiopathic subglottic stenosis had detectable levels of pepsin in their larynx or tracheal mucosa. These results were significant as there was no pepsin found in the control groups. Since pepsin is produced exclusively in the stomach, finding its presence outside of the gastric region points to extraesophageal reflux. Chronic exposure to reflux can result in inflammation, damage, and eventual scarring of the subglottic region [10]. This could point to the idea that acid reflux may contribute to idiopathic subglottic stenosis and should therefore be considered during the evaluation of patients with such symptoms.

Given the wide range of causes for individuals to develop symptoms of shortness of breath, the diagnosis of idiopathic subglottic stenosis is often overlooked. In fact, between one-third and two-thirds of patients with this condition are initially misdiagnosed with asthma or chronic obstructive pulmonary disease (COPD) [5,8,11,12]. Many of these patients diagnosed with asthma or COPD are treated with corticosteroids, which are typically ineffective in this context [8]. In our study, 90% of patients either had been misdiagnosed with asthma or allergies or were treated with inhalers or antihistamines without relief. The exact duration of these prior diagnoses was not documented. Patients in our study had not been misdiagnosed with COPD as other literature has frequently discussed. Similar to other airway disorders, common symptoms of subglottic stenosis include dyspnea on exertion and at rest, stridor, a chronic cough, and occasional dysphonia [8]. Patients in our study presented with similar clinical features. It is typically felt that most patients may remain asymptomatic until the airway is 50% stenosed [5,10,12]. Most patients report a slow and steady worsening of shortness of breath over several months. It may be most noticeable with exertion.

Identification and treatment of idiopathic subglottic stenosis are often delayed. Patients often do not seek out medical attention for an average of six months after the onset of their shortness of breath symptoms [8]. An even greater delay results from initial misdiagnosis. In a case series of 124 patients, Berges et al. reported an average interval of 24.5 months from the onset of symptoms to the diagnosis of idiopathic subglottic stenosis [8]. Other studies have documented even longer delays, with an average time of 36 months from the onset of symptoms to correct diagnosis [13]. Our study reflects a similar trend with an average delay of diagnosis of approximately 36.8 months.

Patients with idiopathic subglottic stenosis are often referred to multiple specialists prior to receiving appropriate otolaryngologic care. Due to these diagnostic delays, some patients ultimately present in respiratory distress and may require an urgent tracheotomy [8].

The potential diagnosis of idiopathic subglottic stenosis can be suspected from the patient's history, physical examination, pulmonary function testing, and flow volume loops. Caucasian females between the ages of 30 and 60 who present with unexplained respiratory symptoms should raise some suspicion of idiopathic subglottic stenosis. Following a thorough history, physical examination may reveal signs of upper airway obstruction. Before any invasive examination, turbulent upper respiratory airflow may be heard. While patients may exhibit this finding at rest, others may require deep inspiration and expiration in order to observe this abnormal airflow. Nine out of our 10 patients had detectable abnormal audible airway sounds at their initial visit.

Pulmonary function tests and flow volume loops are additional tests that can help aid in the evaluation of airway obstruction. In cases of subglottic stenosis, flow volume loops typically demonstrate a fixed upper airway obstruction characterized by flattened and restricted inspiration and expiration loops [14]. In the pulmonary function test, peak expiratory flow is often diminished well below 80% of the predicted value [15]. In our study, the average peak airflow at presentation was 45.4% of the predicted value.

When idiopathic subglottic stenosis is suspected, endoscopy provides the standard for diagnosis of this condition. This can be performed by bronchoscopy, but the stenosis can also be visualized by an otolaryngologist using nasolaryngoscopy [11,12]. Endoscopic evaluation confirms the diagnosis and allows for the assessment of the location and extent of the stenosis [12]. Radiologic imaging, such as a computed tomography scan, may be considered on a case-by-case basis, but is not routinely performed.

Though it is not the focus of this paper, a typical treatment would be a biopsy, followed by laser excision of stenosis, steroid injection, and balloon dilation. There are novel procedures that are undergoing research for a more definitive treatment option for this condition.

Limitations

This study is based on a retrospective cohort that examined 10 consecutive patients who were diagnosed and treated with surgery for idiopathic subglottic stenosis. These patients were collected from a single otolaryngology clinic in Kansas and were selected following the biopsy performed at the time of surgery. This could pose as a limitation as the sample size is small. In addition, potential selection and/or referral bias is possible, as this study may focus on advanced cases and may not represent the greater (and possibly less symptomatic) population.

Many of the preoperative pulmonary function tests for our patients were performed at other clinics, so there could be some variability in testing technique and documentation. This could affect the reproducibility of this information. Though all of our patients did have flow volume loops preoperatively, not all of them had this same testing postoperatively. As this study is directed at diagnosis rather than treatment efficacy, the postoperative testing is of somewhat less importance.

Our study revealed a delay in diagnosis of 36.8 months from symptom onset to diagnosis. We also know that on many occasions, there were significant delays in diagnosis even after the patient had sought out medical attention. Our study does not have enough data to clearly determine the amount of this delay in diagnosis that may have occurred even after initial medical consultation. This would be an interesting and important area of future research.

## Conclusions

The data from this study, along with findings from existing literature, suggest that a thorough history and careful physical examination can significantly expedite the diagnosis of idiopathic subglottic stenosis. In particular, we believe that listening for loud upper airway sounds and turbulent airflow is crucial in guiding clinicians toward this diagnosis. Once suspected, appropriate pulmonary function testing and visualization with endoscopy can be pursued promptly, facilitating earlier intervention. 
